# Rapid diagnosis of pulmonary tuberculosis

**DOI:** 10.11604/pamj.2014.18.141.2295

**Published:** 2014-06-17

**Authors:** José Mauricio Hernández Sarmiento, Natalia Builes Restrepo, Gloria Isabel Mejía, Elsa Zapata, Mary Alejandra Restrepo, Jaime Robledo

**Affiliations:** 1 School of Health Sciences, Universidad Pontificia Bolivariana (UPB), Medellín, Colombia; 2 Bacteriology and Mycobacteriology Unit, Corporación para Investigaciones Biológicas (CIB), Medellín, Colombia

**Keywords:** Cord Factor, Tuberculosis, MGIT, thin layer agar

## Abstract

**Introduction:**

World Health Organization had estimated 9.4 million tuberculosis cases on 2009, with 1.7 million of deaths as consequence of treatment and diagnosis failures. Improving diagnostic methods for the rapid and timely detection of tuberculosis patients is critical to control the disease. The aim of this study was evaluating the accuracy of the cord factor detection on the solid medium Middlebrook 7H11 thin layer agar compared to the Lowenstein Jensen medium for the rapid tuberculosis diagnosis.

**Methods:**

Patients with suspected tuberculosis were enrolled and their sputum samples were processed for direct smear and culture on Lowenstein Jensen and BACTEC MGIT 960, from which positive tubes were subcultured on Middlebrook 7H11 thin layer agar. Statistical analysis was performed comparing culture results from Lowenstein Jensen and the thin layer agar, and their corresponding average times for detecting Mycobacterium tuberculosis. The performance of cord factor detection was evaluated determining its sensitivity, specificity, positive and negative predictive value.

**Results:**

111 out of 260 patients were positive for M. tuberculosis by Lowenstein Jensen medium with an average time ± standard deviation for its detection of 22.3 ± 8.5 days. 115 patients were positive by the MGIT system identifying the cord factor by the Middlebrook 7H11 thin layer agar which average time ± standard deviation was 5.5 ± 2.6 days.

**Conclusion:**

The cord factor detection by Middlebrook 7H11 thin layer agar allows early and accurate tuberculosis diagnosis during an average time of 5 days, making this rapid diagnosis particularly important in patients with negative sputum smear.

## Introduction

Tuberculosis (TB) is a social and public health problem worldwide, affecting more frequently vulnerable populations. The World Health Organization (WHO) estimated on 2009 a global TB incidence of 137 per 100,000 population with 9.4 million cases and 1.7 million of deaths, being the second cause of infection- related death after HIV infection [1]. In Colombia, according to the Social Protection Ministry, the general incidence of TB was 24.3 per 100,000 population with 10,913 cases reported on 2009. In the same year, 1,665 TB cases were reported at the Department of Antioquia and close to 50% of them where located in Medellín city [2]. Performing a rapid and timely diagnosis and improving the sensitivity of the smear is critical to increase the detection rate of patients with respiratory symptoms in order to control the disease [3].

**Figure 1:**
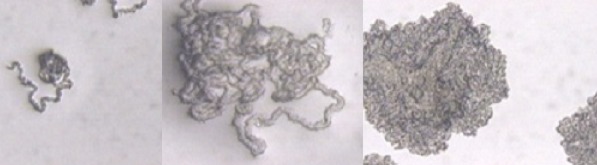
Morphology of the cord factor on the Middlebrook 7H11 thin layer agar subcultured from the MGIT liquid medium, observed with 10X magnification using light microscopy. Microphotograhs kindly provided by Gloria I. Mejía, Bacteriology and Mycobacteriology Unit, Corporación para Investigaciones Biológicas, Medellín, Colombia

Some studies have evaluated the usefulness of cord formation detection, which is named “cord factor” (CF), for the presumptive identification of Mycobacterium tuberculosis complex [4, 5]. The CF is a molecule generated from the glycolipid trehalose 6-6′-dimycolate, a component of the mycobacterial cell wall which is toxic to mammalian cells and affects the host immune system by inhibiting the migration of polimorphonuclear neutrophils. The CF is responsible for the specific microscopic morphology observed in the M. tuberculosis complex growth called serpentine cords [6, 7] (Figure 1). Other microorganisms can also produce this cord, as members of the bacterial genera Nocardia and Rhodococcus, and other mycobacteria such as M. marinum and M. kansassi [8–10]. It has been suggested that the presence of the CF, besides the rough colonial morphology, is associated with virulence of mycobaterial strains and it is one of the most important virulence factors present in M. tuberculosis [8, 11, 12].

The objective of the present study was to evaluate the accuracy of the CF detection on a solid medium as Middlebrook 7H11 thin layer agar as a rapid method for the presumptive diagnosis of TB.

## Methods

All patients underwent medical evaluation before being enrolled in the study. After signing informed consent an average of two sputum samples were taken per patient. If the first sample was smear positive, such specimen was processed to identify M. tuberculosis; if this sample was negative smear, a second specimen was taken for identification of the mycobacterium. Sputum samples were processed for microscopic examination preparing direct smear and staining by auramine-rhodamine staining technique (positive samples were confirmed by Kinyoun staining) at Bacteriology and Mycobacteriology Unit at Corporación para Investigaciones Biológicas, Medellín (Colombia). According to the American Lung Association, acid-fast bacilli (AFB) were quantified on direct smear as follows: negative: AFB not found,+: 3-9 AFB in entire smear, ++: 10 or more AFB in the entire smear, +++: 1 AFB per field [13, 14]. For culture, samples were inoculated into Lowenstein-Jensen (LJ) slant solid medium, and the BACTEC MGIT 960 system (Becton Dickinson, New Jersey, USA). The MGIT was a mycobacteria growth indicator tube with liquid medium that contained 7 ml modified Middlebrook 7H9 broth base, to which an enrichment factor, OADC (bovine albumin, dextrose, catalase and oleic acid) and an antibiotic mixture, PANTA (polymyxin B, amphotericin B, nalidixic acid, trimethoprim and azlocillin), were added. Additionally, MGIT tubes have an oxygen-quenched fluorochrome (tris 4, 7-diphenyl-1, 10-phenonthroline ruthenium chloride pentahydrate) embedded in silicone at the bottom of the tube. If microorganisms are growing, the free oxygen is utilized and is replaced with carbon dioxide, depleting free oxygen so that the fluorochrome is no longer inhibited, resulting in fluorescence within the MGIT tube when visualized under ultraviolet light [15]. The BACTEC MGIT 960 detects such fluorescence as positive signal by emitting an alarm. According to the protocol established in the laboratory, MGIT tubes identified as positives were centrifuged, smears were prepared from such sediments, which were stained by Kinyoun staining and were subcultured on Middlebrook 7H11 thin layer agar, where the CF was identified. The final identification of M. tuberculosis was based on niacin [13, 16], nitrite reduction and catalase tests [13, 14, 16].

Statistical analysis was performed to compare culture results obtained from LJ medium (gold standard) and the Middlebrook 7H11 thin layer agar subcultured from the BACTEC MGIT 960 system, comparing as well their corresponding average times for detecting the mycobacterium or CF in the case of thin layer agar. Another comparison was carried out between direct smear and culture result. In order to evaluate the performance of CF detection as an indicator of M. tuberculosis′ presence, it was determined its sensitivity, specificity, positive predictive value (PPV) and negative predictive value (NPV).

This study was approved by the Ethics Research Committee at Corporación para Investigaciones Biológicas (CIB).

## Results

**Figure 2:**
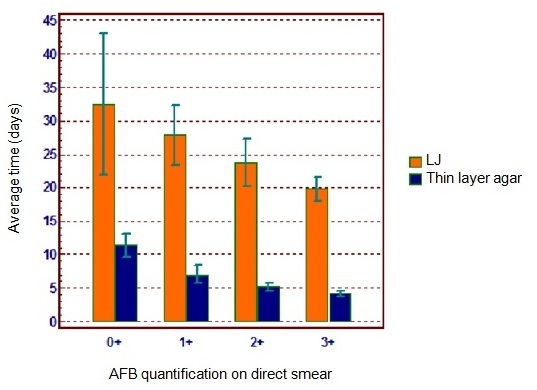
Average time to obtain a presumptive positive result for M. tuberculosis by LJ and Middlebrook 7H11 thin layer agar subcultured from positive MGIT tubes

260 patients were enrolled in the study. 111 (43%) out of them were positive for M. tuberculosis by the LJ medium which average time ± standard deviations (SD) for detecting the mycobacterium was 22.3 ± 8.5 days (Figure 2) and of these, 10 patients were negative by the MGIT system. 115 (44%) patients were positive for M. tuberculosis by the MGIT system being positive as well for CF by the Middlebrook 7H11 thin layer agar subculture, which average time ± SD for CF detection was 5.5 ± 2.6 days (Figure 2) and 14 patients out of them were culture negative by the LJ. 135 patients were negative for both culture methods.

The detection of the CF as an indicator of the presence of M. tuberculosis had a 92.1% sensitivity, 98.5% specificity, 98.3% PPV, 92.9% NPV. The odds ratio for a CF positive test was 61 and for a negative test was 0.08.

## Discussion

The reemerging of the M. tuberculosis infection has renewed the need to increase the initiative for introducing new diagnostic tools with good levels of sensitivity, specificity, low cost and feasibility to be implemented in resource-limited settings [17–19]. Our study demonstrated that the CF detection by Middlebrook 7H11 thin layer agar, the solid medium used to subculture positive MGIT tubes, as diagnostic method for pulmonary TB in patients with respiratory symptoms from Medellín (Colombia), had a sensitivity of 92.1% and a specificity of 98.5%, allowing a presumptive report of the isolation of M. tuberculosis in 90% of the cases during an average time of 5 days. This period of time may vary depending on the patient's bacteria load, being up to 10 days for patients with negative smear and positive culture, and up to 4 days for patients with one AFB per field on direct smear.

Other studies have suggested the usefulness of CF detection for rapid identification of the mycobacterium. One of them was carried out by Brazilian researchers from the Adolfo Lutz Institute, where 743 M. tuberculosis clinical strains were analyzed, obtained from patients with respiratory symptoms. In such study, it was found that the CF detection in liquid and on solid medium had a sensitivity of 98.5%, specificity of 88%, PPV of 97% and NPV of 93% compared to conventional typing tests, morphological analysis of growth and traditional biochemical methods, concluding that identifying cord growth is a rapid criterion for the identification of the M. tuberculosis complex [5]. Another study performed in the same Brazilian institute compared the detection of the CF with the PCR-restriction enzyme analysis (PRA) as the gold standard, suggesting that the presumptive identification of M. tuberculosis using the macroscopic evaluation of colonies combined with CF detection under microscopy is a simple, rapid and inexpensive test to be recommended in resource-poor settings [20].

Although is important to mention that an experienced technician is needed to appropriately identify the cord formation, the equipment and training for such a goal are cheaper and easier than those needed to apply other technologies such as molecular biology techniques, supporting resource-limited settings and less well-equipped laboratories [20, 21]. The CF detection has proved to be a simple and rapid test that requires only a good microscopy technique and simple culture systems, solid or liquid medium, for detecting M. tuberculosis [22, 23].

## Conclusion

The present study suggests that the CF detection is a simple and rapid test, demonstrating a sensitivity of 92.1% and specificity of 98.5% leading to the identification of M. tuberculosis in only 5 days. Rapid diagnosis is particularly important in patients with negative sputum smears, because of the risk of this group of patients to undergo delays during diagnosis process and subsequent treatment.
